# Enzyme Conditioning of Chicken Collagen and Taguchi Design of Experiments Enhancing the Yield and Quality of Prepared Gelatins

**DOI:** 10.3390/ijms24043654

**Published:** 2023-02-11

**Authors:** Pavel Mokrejš, Robert Gál, Jana Pavlačková

**Affiliations:** 1 Department of Polymer Engineering, Faculty of Technology, Tomas Bata University in Zlín, Vavrečkova 275, 760 01 Zlín, Czech Republic; 2 Department of Food Technology, Faculty of Technology, Tomas Bata University in Zlín, Vavrečkova 275, 760 01 Zlín, Czech Republic; 3 Department of Lipids, Detergents and Cosmetics Technology, Faculty of Technology, Tomas Bata University in Zlín, Vavrečkova 275, 760 01 Zlín, Czech Republic

**Keywords:** biomaterials, by-product, enzyme conditioning, collagen, gelatin, mechanically deboned chicken meat, Taguchi design, zero-waste

## Abstract

During the production of mechanically deboned chicken meat (MDCM), a by-product is created that has no adequate use and is mostly disposed of in rendering plants. Due to the high content of collagen, it is a suitable raw material for the production of gelatin and hydrolysates. The purpose of the paper was to process the MDCM by-product into gelatin by 3-step extraction. An innovative method was used to prepare the starting raw material for gelatin extraction, demineralization in HCl, and conditioning with a proteolytic enzyme. A Taguchi design with two process factors (extraction temperature and extraction time) was used at three levels (42, 46, and 50 °C; 20, 40, and 60 min) to optimize the processing of the MDCM by-product into gelatins. The gel-forming and surface properties of the prepared gelatins were analyzed in detail. Depending on the processing conditions, gelatins are prepared with a gel strength of up to 390 Bloom, a viscosity of 0.9–6.8 mPa·s, a melting point of 29.9–38.4 °C, a gelling point of 14.9–17.6 °C, excellent water- and fat-holding capacity, and good foaming and emulsifying capacity and stability. The advantage of MDCM by-product processing technology is a very high degree of conversion (up to 77%) of the starting collagen raw material to gelatins and the preparation of 3 qualitatively different gelatin fractions suitable for a wide range of food, pharmaceutical, and cosmetic applications. Gelatins prepared from MDCM by-product can expand the offer of gelatins from other than beef and pork tissues.

## 1. Introduction

Gelatin is one of the most versatile biopolymers, and due to its unique film, gel, and surface properties, it is widely used in the food, pharmacy, cosmetics, and photography industries, as well as in the production of packaging materials and encapsulates and in a number of technical applications [1,2,3]. This is evidenced by the global production of gelatin, which represented approximately 700 kilotons in 2021; the total turnover in terms of raw material represents approximately 3500 million USD. Of this amount, approximately 30% was consumed in the production of food and beverages, 25% in nutraceuticals, 19% in pharmaceuticals, 14% in photography, 7% in personal care products, and 5% in other applications. A further increase in gelatin production is expected for 2025 by approximately 6.0% compared to 2019 [4]. Gelatin can be made from any animal tissue that contains collagen. Currently, approximately 95% of all gelatin is produced industrially from beef and pork tissues. The rest consists of alternative sources of collagen which have gained importance in the last 20 years not only due to the growing demand for gelatin but also due to special consumer requirements [5]. Some examples are religious or cultural reasons for rejecting pork or beef products or consumer preferences for fish or poultry products over beef and pork. It is also necessary to mention the socially changing attitudes towards handling animal by-products and the possibilities of their use (philosophy of the circular economy). Gelatins can be prepared from various unused parts of poultry, most commonly chicken feet and skin [6,7], duck feet and skin [8,9], and chicken bones [10,11]; other types of poultry are less common [12]. From fish (both freshwater and marine), gelatins are most often prepared from skin, bones, scales, fins, or heads [13,14,15,16,17]. The conditions for preparing gelatin from frog skin are also known [18]. However, the disadvantage of alternative raw material sources containing collagen is their non-standard parameters, which significantly complicates their processing into gelatins with properties suitable for specific applications. For example, gelatins prepared from cold-water fish species are less stable and have worse rheological properties, which also complicates their processing. There are also fundamental differences in the properties of gel formation (gel strength, gelling, and melting point) between gelatins prepared from cold and warm water fish [19,20,21,22].

When processing collagen raw materials (mainly skin and tendons) into gelatins, it is necessary to remove accompanying components (most often fat, globular proteins, and glycoproteins) from the starting raw material and to prepare the raw material in a suitable way for controlled extraction. For this purpose, traditional or alternative procedures are used—namely conditioning in an acidic or alkaline environment and rarely the use of enzymes [2,23]. The exception is for bones for which demineralization is necessary. This is done in an acidic environment [24]. Gelatin extraction is carried out with hot water (depending on the type of raw material at a temperature of 40 °C minimum) in extractors of various designs. In the industrial production of beef and pork gelatin, multistage extraction is used to efficiently convert collagen into gelatin [2].

Mechanically deboned meat can be obtained from all animals, with the exception of ruminants, which have been banned as a raw material since 2011 due to concerns about the possible disease of bovine spongiform encephalopathy (BSE). Mechanically deboned chicken meat (MDCM) is obtained most often and used for the production of meat products [25,26]. It is obtained by mechanical separation of the remaining parts of the meat, which are found in the bones and ribs after the meat has been cut and can make up to 30% of the muscle content. To obtain MDCM, a traditional separation procedure is used, which is based on pressing bone raw materials; a continuous filling and pressing method or a separate filling and pressing process can be applied. During the pressing technique, the muscle with the fatty and connective parts is separated from the bones and rough connective tissues. When the MDCM separation decantation procedure is applied, bone raw materials are ground with the addition of flake ice and a sodium nitrite curing salt mixture. The resulting liquid homogenate is continuously centrifuged based on the principle of decantation and immediately frozen. Screw conveyors, hydraulic pistons, or drum separators are used for separation; the yield and quality of MDCM can be regulated, for example, by the size of the holes in the separation sieves or the flow rate of crushed meat and bone raw material [27]. In the MDCM separation process, higher pressures are sometimes used to increase the yield, resulting in a higher Ca content in the MDCM. Due to the presence of a higher amount of mineral substances, MDCM has good water binding capacity and is suitable as an addition to sausages, pâtés or poultry semi-products; additions up to 10% do not negatively affect the properties of the final products [28,29]. MDCM has a limited shelf life, which is related to the possibility of microbial contamination, the increase in temperature during the separation process, and the higher pH value due to the Ca_3_(PO_4_)_2_ content. The solid residue after MDCM production is characterized by a high content of proteins (up to 40% in dry matter), fats (25–30% in dry matter), and minerals (approximately 30% in dry matter) and thus represents an important source of raw materials rich in nutrients.

In addition to the basic physicochemical properties of gelatin (composition, swelling, solubility, color, clarity, odor, and taste), the main attributes that best define the commercial quality of gelatin include gel strength and viscosity [30]. However, the complex quality of gelatins is determined by a set of gel-forming and surface properties. These are important not only for the application of gelatin in final products but also for the choice of a suitable processing technology (extrusion, casting, dipping, injection). The gel-forming properties also include the gelling point (GP), melting point (MP), water holding capacity (WHC), and fat binding capacity (FBC). Surface properties include foaming capacity (FC) and foaming stability (FS), emulsifying capacity (EC), emulsion stability (ES), film-forming ability, and adhesive and cohesive properties. The properties of gelatins depend on many factors, especially the type of collagen (beef, pork, fish, poultry), the conditions of collagen processing (acidic, alkaline, enzyme, combined), the conditions of gelatin extraction (especially temperature, pH, time), and the methods of processing the extracted gelatin (especially the choice of drying method) [31]. The type of collagen and the processing conditions affect the amino acid composition of gelatin and the distribution of molecular weights [32,33]. The representation and ratio between α-, β-, and γ-chains in gelatin affects the viscosity of gelatin (viscosity increases as the amount of β- γ-chains increases) [34]; it also affects the GP and MP of gelatin (a higher representation of α-chains shifts both temperatures to higher values) [31]. The structural stability of gelatin is mainly due to the content of the amino acids proline and hydroxyproline, which contribute to the stabilization of the structure by means of hydrogen bridges [35]. A higher content of these amino acids will be reflected in an increase in the GP and MP of gelatin [36]. More detailed information on the structure of gelatin is provided by rheological measurements [37], scanning and transmission electron microscopy (SEM and TEM) [38], Fourier transform infrared (FTIR) spectroscopy [39], and differential scanning calorimetry (DSC) [40].

In our previous study devoted to the preparation of gelatin from the MDCM by-product, a two-level factorial experiment with three studied process factors was used [41]. Compared to studies devoted to the preparation of gelatin from the same raw material [42,43,44], in our work higher gelatin yields were achieved. In our study, the basic properties of gelatin (gel strength, viscosity, ash content) were determined. It is clear that all studies showed important results in regard to the processing of previously unused MDCM by-products into gelatins. Considering the great potential of this raw material source, it would be advisable to deal with a more detailed optimization of the gelatin preparation procedure, a thorough characterization of gelatin, and the proposal of its applications with regard to their properties.

The objectives of the current study are as follows: (1) Optimize the process of preparing gelatin from the MDCM by-product to achieve the maximum degree of conversion of the starting raw material to gelatins without a negative effect on their quality. For this purpose, we propose an innovative process and Taguchi design of experiments: demineralization of the MDCM by-product, enzyme conditioning of the purified collagen, and 3-stage gelatin extraction; (2) design the processing technology so as to limit the number of by-products created; (3) perform a comprehensive assessment of the quality of prepared gelatins by determining their gel-forming and surface properties; (4) propose potential industrial applications of the prepared gelatins. Scientific hypotheses: By adjusting the process conditions during the processing of collagen from MDCM by-product into gelatins, gelatins are prepared with a higher yield than using standard technological procedures. The higher yields of gelatin will not have a negative effect on their properties.

## 2. Results

The results of processing the MDCM by-product into three fractions of gelatins are presented in the following four subsections.

### 2.1. Mass Balance of the Process

The schedule of experiments and results of the processing of the MDCM by-product into three gelatin fractions are presented in Table 1. Table 2 shows the results of the analysis of variance for the gelatin yields.

**Figure 1:**
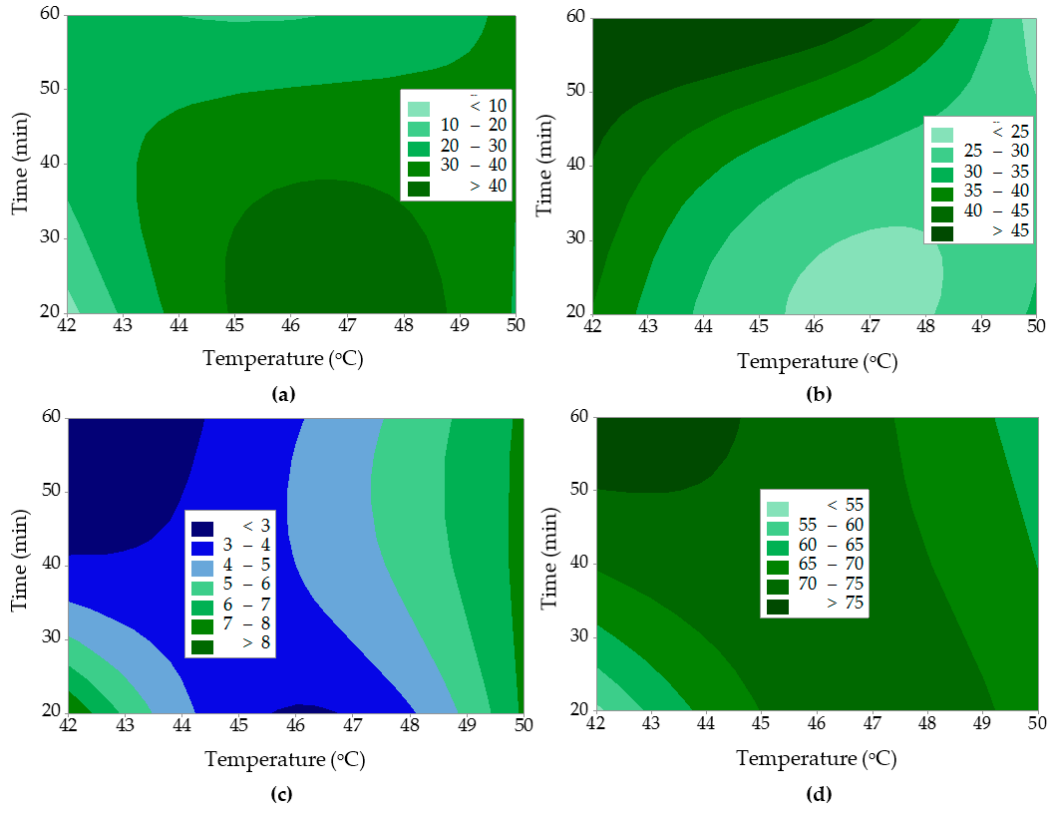
The influence of extraction temperature at 1st extraction step and extraction time at 1st extraction step on gelatin yields: (**a**) the yield of the 1st gelatin fraction; (**b**) the yield of the 2nd gelatin fraction; (**c**) the yield of the 3rd gelatin fraction; (**d**) the total yield of gelatins.

Figure 1 shows the relationship between a response variable (gelatin yields) and two predictor variables (extraction temperature and extraction time) using contour plots. Depending on the values of both studied process factors, the yield of the first gelatin fraction (Y_G1_) ranges from less than 10% to more than 40%. The highest Y_G1_ yields were achieved at extraction temperatures of 45–49 °C with extraction time < 35 min (see Figure 1a); both studied process factors were not found to be significant at the monitored level of significance (*p*-value ≤ 0.05). The second gelatin fraction (Y_G2_) is among the dominant gelatin fractions in terms of percentage representation, with yields of approximately 22 to 50%; gelatins from the second fractions show the best gel-forming and surface properties (see Section 2.3). From Figure 1b, there is an obvious trend of Y_G2_ yield growth, especially with increasing extraction time (Factor B). The extraction time is a statistically significant factor with a *p*-value = 0.024, see Table 1. On the contrary, it is evident from the contour position that the extraction temperature (Factor A) has a smaller effect on Y_G2_; the *p*-value is higher than 0.05. The third gelatin fractions, with their approximate yield (Y_G3_) of 2–8%, have the lowest representation of extracted gelatins, see Figure 1c. Neither of the two monitored process factors is statistically significant (*p*-values are > 0.05). Figure 1d then shows the total yield of extracted gelatin, Y_G**∑**_ (sum of Y_G1_, Y_G2_, and Y_G3_). It is obvious that at an appropriately chosen extraction temperature (42–44 °C) and an extraction time of 50–60 min, the degree of collagen-to-gelatin conversion is very high, up to approximately 75%.

If we compare the yields of gelatins (Y_G1_, Y_G2_, and Y_G3_) prepared according to our proposed procedure consisting of demineralization of the starting raw material, enzyme conditioning of collagen, and 3-stage gelatin extraction according to Taguchi design (see Exp. Nos. 1–9 in Table 1) with a blind experiment under conditions corresponding to the mean values of the monitored factors (extraction temperature 46 °C and extraction time 40 min) without enzyme collagen conditioning (see Exp. No. 10 in Table 1), it is evident that the innovative method of collagen conditioning has a fundamental effect on gelatin yield. The total yield of gelatin (Y_G**∑**_) in the blind experiment is only 8.1%, which is approximately 9 times less than that for gelatin extracted under the same process conditions (Exp. No. 5) with enzyme collagen conditioning. Compared with the yield of gelatins prepared under different conditions (Exp. Nos. 1–9), the Y_G**∑**_ in the blind experiment is 6.7–9.5 times lower.

### 2.2. First Gelatin Fractions

The results of the properties analysis of the first gelatin fractions prepared from the MDCM by-product are shown in Table 3.

None of the gelatins obtained in the first extraction step formed measurable gels; therefore, these are zero Bloom value gelatins. The zero Bloom value is also related to the viscosity of gelatin, which reaches very low values (1.4–1.7 mPa·s), regardless of the changing extraction conditions. Similarly, it is with WHC, where no significant difference between gelatins is apparent; depending on extraction conditions, WHC = 220–250%. For FBC, a slight growth trend is evident with increasing extraction temperature and, at the same time, prolonging extraction time; from values slightly exceeding 800% at the minimum values of both monitored factors to approximately 1200% at the upper limits of the factors. Foaming properties, FC and FS, are very low, 6 to 8% or 0 to 4%, respectively; temperature and extraction time do not fundamentally affect these parameters. It is similar to the emulsifying properties, EC and ES, for which process conditions do not affect their changes. However, all gelatins have very good EC values (46–48%) and excellent ES (92–95%). The properties of gelatin prepared under the conditions of a blind experiment (without enzyme conditioning) under conditions corresponding to the mean values of the monitored factors (extraction temperature 46 °C and extraction time 40 min)–see Exp. No. 10 in Table 3–do not fundamentally differ from the properties of gelatin prepared in Exp. Nos. 1–9.

### 2.3. Second Gelatin Fractions

The results of the properties analysis of the second gelatin fractions prepared from the MDCM by-product are shown in Table 4. Table 5 shows the results of the analysis of variance for the strength of the gelatin gel, the viscosity, the melting point, and the gelling point.

The ash content is very low in all gelatins prepared according to the Taguchi design (Exp. Nos. 1–9); it varies between 0.34–0.70%. Fundamental differences were not found in the water holding capacity (930–1090%) and fat binding capacity (980–1470%). Gelatin prepared according to the conditions of Exp. No. 9 has a significantly higher foaming capacity (36%) than the other gelatins (18–22%); there is a similar difference in foaming stability (24% versus 8–18%). In terms of emulsifying capacity and emulsion stability, there are no fundamental differences between the gelatins prepared according to experiments 1–9.

Properties of gelatin prepared under the conditions of a blind experiment (without enzyme conditioning) under conditions corresponding to the mean values of the monitored factors (extraction temperature 46 °C and extraction time 40 min)–see Exp. No. 10 in Table 4–differs significantly in some parameters from the properties of the gelatins prepared in Exp. Nos. 1–9. In particular, this is a very high gel strength value, which is 1.6 to 5.8 times higher compared to gelatins prepared from Exp. Nos. 1–9; for viscosity, the value is 1.8–4.3 times higher. WHC (1320% versus 930–1090%) and FBC (1540% versus 980–1470%) are also higher. This is also true for FC (40% versus 18–36%) and FS (28% versus 8–24%). There are no fundamental differences in EC and ES for gelatin from Exp. No. 10 compared to gelatins prepared according to Exp. Nos. 1–9.

**Figure 2:**
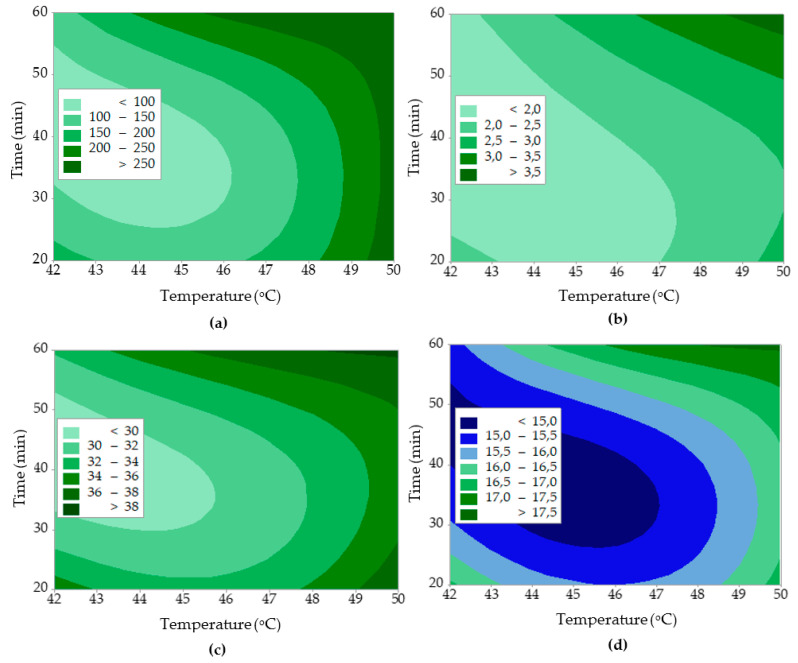
The influence of extraction temperature and extraction time on second gelatin fractions properties: (**a**) gel strength; (**b**) viscosity; (**c**) melting point; (**d**) gelling point.

Figure 2 shows the relationship between the response variables and two predictor variables (extraction temperature and extraction time) by contour plots. From Figure 2a, the trend of increase in gel strength is evident, especially with increasing extraction temperature; extraction temperature is a statistically significant factor (*p*-value of 0.016; see Table 5). Lower gel strength values (up to 200 Bloom) are achieved at temperatures < 48 °C and extraction times up to 50 min. Very good gel strength values (200–250 Bloom) are achieved at extraction temperatures close to the upper limit of the observed temperature (50 °C), while the extraction time does not have a significant effect on the gel strength value. A very similar trend of influence of extraction temperature and extraction time on gelatin viscosity can be seen in Figure 2b. Gelatins with a lower viscosity (2.0–2.5 mPa·s) are prepared at an extraction temperature <42.5 °C regardless of the extraction time; increasing the extraction temperature to 50 °C while simultaneously shortening the extraction time has the same effect. The highest viscosity (3.0–3.5 mPa·s) was achieved at extraction temperatures of 49–50 °C with extraction times >55 min. The extraction temperature is a statistically significant factor (*p*-value = 0.021), see Table 5. An almost identical effect of both process factors, as with gel strength, was recorded on the MP; see Figure 2c. The melting point ranges from relatively lower values (around 30–32 °C) at lower extraction temperatures (<47 °C) without a significant influence on extraction time. A very high MP (35–38 °C) is achieved at extraction temperatures above 49 °C; the extraction time has no significant effect on the change in MP values. The GP is not fundamentally affected by changes in the monitored process conditions; it ranges from 15.0 to 17.5 °C, with lower GP values corresponding to lower extraction temperatures and shorter extraction time, and higher GP values to extraction temperatures >49 °C. Both monitored process factors are statistically insignificant (*p*-values > 0.05, see Table 5).

### 2.4. Third Gelatin Fractions

The results of the properties analysis of the third gelatin fractions prepared from the MDCM by-product are shown in Table 6.

From the results of the third gelatin fraction properties, gelatins prepared under Taguchi design conditions (Exp. Nos. 1–9) can be divided into 3 groups; the first group consists of gelatins prepared at the lowest extraction temperature (42 °C, Experiments 1–3), the second gelatins prepared at medium extraction temperature (46 °C, Experiments 4–6) and the third gelatins prepared at the highest extraction temperature (50 °C, Experiments 7–9); see Table 6. The most fundamental is the difference in the strength of the gels. While gelatins prepared at 46 °C did not form gels at all and gelatins prepared at 42 °C formed weak gels (80–88 Bloom), gelatins prepared at 50 °C had very high gel strengths (223–230 Bloom). The differences between MP and GP are not fundamental between gelatins with the ability to form gels. However, for gelatins prepared at 50 °C, the MP (33.9–34.8 °C) is higher than for gelatins prepared at 42 °C (29.2–30.8 °C); for GP, there is a difference between these two groups of gelatins, 16.0–16.5 °C versus 14.9–15.3 °C. The group of gelatins prepared at 46 °C did not form gels; therefore, it was not possible to determine MP and GP for these gelatins. For viscosity, the trend is analogous to that of gel strength; the highest (2.4–2.6 mPa·s) in gelatins prepared at 50 °C, followed by gelatins prepared at 42 °C (1.7–1.8 mPa·s), with a slight decrease in gelatins prepared at 46 °C. The ash content of all 9 prepared gelatins is very low and ranges from 0.48 to 0.96%. The water holding capacity is 2.6 to 3.2 times lower for gelatins prepared at 46 °C than for gelatins prepared at 42 °C and even 3.2 to 3.5 times lower than for gelatins prepared at 50 °C; 210–220% versus 550 to 680% versus 680 to 730%. There are no significant differences in FBC between the three groups of gelatin; FBC = 990–1220%. On the other hand, in FC, gelatins prepared at 46 °C outperform both gelatins prepared at 50 °C (18–20% versus 16–17%) and gelatins prepared at 42 °C, which have a very low FS (6–8%). For gelatins prepared at 42 and 50 °C, there is zero FS, while for gelatins prepared at 46 °C, it is 11–12%. There are no significant differences in EC and ES between gelatins prepared according to experiments 1–9.

The properties of gelatins prepared under the conditions of a blind experiment (without enzyme conditioning) under conditions corresponding to the mean values of the observed factors (extraction temperature 46 °C and extraction time 40 min)–see Exp. No. 10 in Table 6–are comparable to gelatins prepared according to Exp. Nos. 7–9. In particular, the gel strength (245 Bloom), WHC (910%), and FBC (1220%) are slightly higher.

## 3. Discussion

### 3.1. Comparing and Contrasting Results with References

Our previous study [41] and sources available in the literature [42,43,44] on the processing of MDCM by-products into gelatins and selected literary sources describing the processing of other alternative collagen tissues into gelatins [8,18,45,46,47] were selected to compare and contrast the results achieved in our current study.

#### 3.1.1. Technological Conditions for Gelatin Preparation and Gelatin Yield

In Table 7, for comparison, the summary of key technological operations and gelatin preparation conditions is presented according to our current study and the comparative studies, including the results of the gelatin yields [8,18,41,42,43,44,45,46,47].

When directly comparing gelatin yields from the MDCM by-product with literature describing the processing of the same raw material, it is clear that gelatin yields are primarily influenced by the method of collagen conditioning and the extraction temperature. On the other hand, the highest yields of gelatin (15–17%, see Table 7) are essentially unaffected by the method of conditioning the raw material (acidic or alkaline) [42,43,44]. The preparation of gelatin according to Rafieian et al. [42] seems to be the simplest process; after separating the fat and accompanying non-collagenous matter from the raw material, the acid conditioning of the raw material directly proceeds; subsequently, after washing and neutralization, gelatin (with a yield of up to 17%) is extracted. As a result of the missing demineralization step, it was necessary to subject the prepared gelatins to ion exchange. On the contrary, Rammaya et al. [43] and Erge and Zorba [44] applied a procedure common to the production of pig and bovine bone gelatins consisting of the demineralization of bones in an acidic environment and then the conditioning of collagen in an alkaline environment followed by the extraction of gelatin with hot water. Through extraction under different process conditions, a gelatin yield of up to 15–16% was achieved. In direct comparison with our previous study [41], it is evident that in the current study, including the demineralization process and optimizing the technological process of gelatin extraction, consisting of adjusting the temperatures and extraction times and increasing the number of extraction cycles, there is an obvious increase in the total gelatin yield from 23.2–38.6% [41] to 53.9–76.8% (current study).

Camel bone collagen, although with a significantly higher degree of intermolecular crosslinking than the MDCM by-product collagen, after demineralization in HCl and acid conditioning in the same type of acid, was processed into gelatin with a significantly lower efficiency (8.5–25.3%) [45] than was the case in our current study, where the total gain of the three gelatin fractions was 53.9–76.8%. Compared to collagen of a similar type, duck skin [8], our gelatin preparation technology shows higher yields than gelatin preparation with four different extraction methods (11.7–44.0%) studied by Kim et al.; see Table 7. Gelatin production was significantly lower (2.2–15.4%) in chicken, frog, and tuna skins [18]. This is very surprising considering the same or very similar type of collagen. Shyni et al. [46] recorded similar extraction yields (11.3–19.7%), and their study investigated the possibilities of extracting gelatin from tuna, shark, and rohu skin after conditioning collagen with diluted CH_3_COOH. Therefore, it can be concluded that the reason is most likely the chosen method of conditioning, as the other technological steps of gelatin preparation are similar in our and compared studies. Therefore, it is evident that the enzyme method of purified collagen processing results in higher gelatin yields. On the other hand, compared to the study [18] with lower gel strength values, and compared to [46] with higher gel strength, see Section 3.1.2. It is interesting to compare with the results of the 2-step extraction efficiency of gelatin from bovine heart collagen [47], which can be assumed to have a lower degree of intermolecular crosslinking than chicken bone collagen. After the first gelatin extraction, in which the starting raw material was not conditioned, the gelatin yield was very low (7–11%, see Table 7), due to the weak disruption of the collagen quaternary structure. After the second gelatin extraction, which was preceded by conditioning of the raw material in an acid environment (CH_3_COOH) with the combination of enzyme, there was a significant increase in gelatin yields (66–85%) at the expense of their quality parameters; see Section 3.1.2.

#### 3.1.2. Gel Strength, Meting Point and Gelling Point

The strength of gelatin gel is a key property of gelatin that affects its use in industrial practice (food, pharmacy, medicine, cosmetics, photography, etc.). Information on the MP and GP values of pig and bovine gelatins is known and is mostly proportional to the gel strength; as the gel strength increases, MP and GP also increase [2]. For gelatins prepared from alternative collagen tissues (poultry and fish), the MP and GP values are mostly unknown, or their values differ significantly. In fish gelatins, for example, MP is very low and usually varies, depending on the type of fish tissue and its occurrence (warm and cold water fish), mostly between 13 and 22 °C [21]; some authors report even lower values (around 5–6 °C) [19]. The GP of most fish gelatins reaches 16–29 °C [19,22].

Gelatins prepared according to the technological procedure of the current study belong to the category of zero, low-, medium-, and high-Bloom value gelatins. The gelatins of the first fractions did not form gels, the gelatins of the second fractions formed gels with a Bloom strength of 80–290 Bloom (depending on the temperature and extraction time), and the gelatins of the third fractions formed gels with a Bloom strength of 0 to 230 Bloom. In a direct comparison of the gel strength values of gelatins prepared from the same raw material, most of the gelatins prepared by us are significantly higher quality than those prepared according to Rammaya et al. [43], whose gelatins with a gel strength of about 62 Bloom belong to the category of low Bloom value gelatins. In contrast, gelatins prepared from the MDCM by-product according to procedures [42] and [44] have very high gel strengths, reaching at least the highest values for our gelatins, 320–570 Bloom [42] and 281–1176 Bloom [44]. In this context, it is necessary to note that the high gel strength in the study [42] was achieved with significantly lower gelatin yields (1.6–16%) compared to the sum of the gelatin yields of our second and third fraction (30–54%). It is similar compared to the study [44], where the yields of the prepared gelatins were 3.4–5.0 times lower. Compared to our previous study, in which we processed MDCM by-product into gelatins without prior demineralization of the raw material [41], there was up to a 2-fold increase in gelatin yield in our current study. Of the studies mentioned above [42,43,44], only Erge and Zorba [44] tested the MP and GP of gelatins. Our gelatins from the second and third fractions, with values of 28.9–38.4 °C and 29.2–34.8 °C, have similar or slightly higher MP than the gelatins prepared by Erge and Zorba (30.0–33.7 °C). On the contrary, our gelatins have lower GP (14.9–17.6 °C for the second fraction and 14.9–16.5 °C for the third fraction) than the gelatins of the comparative study (18.5–22.5 °C).

Al-Kahtani et al., under optimal processing conditions for camel bone collagen, prepared gelatins with a gel strength of 206 Bloom [45]. Very high gel strength values (210–260 Bloom) were achieved for gelatins prepared from duck skin using various extraction methods (see Table 7) [8]. The gelatins were characterized by good MP values (31.3–33.9 °C), which were not significantly affected by the type of extraction method. Under certain preparation conditions, our gelatins reach higher MP values (up to 38.4 °C). As a result of favorable conditions for conditioning the starting raw material (weak acid solutions) and especially low extraction temperatures (45–65 °C), gelatin prepared from tuna, frog, and chicken skins had excellent gel strength values (336–363 Bloom) [18]. Furthermore, these gelatins show excellent values of MP (30–43 °C) and GP (22–28 °C), which surpasses the gelatins prepared by us (maximum values 38.4 °C and 17.6 °C, respectively). Acid conditioning (0.2 mol/l CH_3_COOH) of selected skin tissues and gelatin extraction at 45 ° C were used in the study by Shyni et al. [46]. Lower gel strength values were achieved for gelatins from rohu (124 Bloom) and tuna (171 Bloom) skins. Consequently, there were very low MP (18.2 °C) and GP (13.8 °C) for rohu skin gelatin; for tuna skin gelatin the values were much higher (24.2 °C and 18.7 °C, respectively). On the contrary, for shark skin gelatin, the gel strength value reached > 200 Bloom with slightly higher MP (25.8 °C) and GP (20.8 °C) values. Therefore, it is clear that the type of collagen affects not only the yield of prepared gelatins (see Table 7), but also their properties. Compared to gelatin prepared from these different types of skins, our gelatins have a higher MP (28.9–38.4 °C); GP is more or less comparable (14.9–17.6 °C). Similar results are obtained in comparison with a study that processed bovine hearts into gelatin in two phases. Gelatins prepared after the first extraction stand out for their very good gel strength (241–269 Bloom); very good values were shown by MP (32.7–33.4 °C) and GP (24.3–25.7 °C). In contrast, gelatins prepared after the second extraction show significantly worse gel-forming properties, gel strength 54–96 Bloom, MP 24.3–27.5 °C, and GP only 14.0–18.5 °C [47].

For pharmaceutical gelatin applications with the highest quality requirements (production of hard gelatin capsules), gelatins with a gel strength in the range of approximately 200 to 280 Bloom are required. For some food applications, e.g., the production of extruded marshmallows, sweet desserts, reduced-fat butter-type spreads, panna cotta or aspics, gelatins with a gel strength of approximately 230 to 270 Bloom are preferred. These gelatins can be prepared according to our proposed technology at an extraction temperature of 50 °C and an extraction time of 20–60 min; gelatin yields are then 30–38%. If we compare the gel strength of the prepared gelatins with the conditions of their preparation (demineralization, enzyme conditioning, low extraction temperature, and very short extraction time) and with the achieved yield, it is clear that the gelatins prepared from MDCM by-product according to our optimized technology surpass the previously published procedures [42,43,44] and the results of our previous study [41] when processing the same initial raw material, but without the demineralization step. The strength of the gels is then comparable to gels prepared according to a number of studies [8,45,46,47]; only gelatins from tuna, frog, and chicken skin had higher gel strengths [18].

#### 3.1.3. Viscosity, Ash, Water Holding Capacity and Fat Binding Capacity

Gelatin viscosity is a key parameter, especially for the choice of a suitable processing technology for gelatin or food recipes containing gelatin. The ash content is a strictly monitored parameter of gelatins for their applications in the food industry, nutritional products, in the production of pharmaceutical capsules, medicine, cosmetics; very strict limits also apply to photographic applications [48,49]. The binding capacities of water and fat are important, above all, for some food applications [2,48,49].

The gelatins of the second fraction with viscosity values of 1.6–3.8 mPa·s belong to the category of low-medium-viscosity gelatins; gelatins of the first and third fractions belong to the category of low-viscosity gelatins (1.4–1.7 mPa·s and 1.4–2.6 mPa·s, respectively). Compared to our previous study, where gelatins with low viscosity values (1.4–2.8 mPa·s) were prepared [41], the viscosity of the second fraction of gelatin prepared under the conditions of experiment 9 increased to 3.8 mPa·s. From available studies on the processing of MDCM by-products into gelatins [42,43,44], the viscosity of prepared gelatins was tested only by Rafieian et al., whose gelatins belong, with values of 2.8–5.8 mPa·s, to medium-high-viscosity gelatins [42]. A high viscosity was found in duck skin gelatin, according to the extraction procedure method it was 5.69–7.79 mPa·s [8]. Similar values were achieved for chicken skin gelatin (7.5 mPa·s) and tuna skin gelatin (5.0 mPa·s); a very high viscosity was found in frog skin gelatin (14.1 mPa·s) [18].

Of the compared studies, only some tested gelatins for WHC and/or FBC [18,46,47]. High WHC values were found for gelatin from frog and chicken skins (1480% and 650%, respectively) [18]. The same study reports the highest value of FBC (3950%) for gelatin prepared from tuna skin, lower (1880%) for frog skin gelatin, and the lowest (229%) for chicken skin gelatin. Bovine heart gelatins showed almost identical WHC values as frog and chicken skin gelatins [18], depending on the method of gelatin preparation, it was 685–1045%; very good values were also found for FBC (783–4373%) [47]. This can be influenced primarily by the type of collagen and the conditions of collagen preparation, which affect the degree of chemical and thermal denaturation of collagen. Slightly different conditioning procedures (weaker acid) and the type of processed collagen resulted in lower WHC values for shark skin gelatin (256%), tuna skin gelatin (214%), and rohu skin gelatin (163%). The FBC of the prepared gelatin also varied (347–452%) [46]. The gelatins prepared in our study with their WHC and FBC are comparable to the gelatins from the above studies. The WHC of our gelatins of the first, second, and third fractions was 220–250%, 930–1090% and 210–730%; FBC then 840–1210%, 980–1470% and 990–1220%.

#### 3.1.4. Foaming Capacity, Foaming Stability, Emulsifying Capacity, and Emulsion Stability

Knowledge of FC and FS helps to optimize recipes for confectionery production (especially for chewy candies and marshmallows), while EC and ES are important properties of gelatins added to meat products [2]. The foaming capacity was highest for gelatin prepared from chicken skin (190%), lower for gelatin from frog skin (143%) and lowest for gelatin from tuna skin (46%) [18]. The values are significantly higher than for the gelatin prepared by us; in the gelatins of the first fraction, the FC was very low (6–8%), in the gelatins of the second fraction it increased to 18–36% and in the third fraction it decreased to 6–20%. The same study reports that FS is approximately half that of FC, which was also found for our gelatins. An example of how the chosen method of conditioning the raw material and the extraction conditions (especially temperature and time) affect the surface properties of the prepared gelatins is demonstrated by the results of another comparative study devoted to the preparation of gelatins from tuna, shark and rohu skins [46]. The FC of these gelatins is low (17.4–21.5% depending on the type of raw material) and corresponds to our gelatins. The foaming stability of gelatin is similar to our second fraction of gelatin.

Gelatins prepared from different types of skin (tuna, frog, and chicken) [18] have comparable EC values (42–69%) as our gelatins (approximately 50%). The ES (depending on the type of gelatin 47–68%) is worse than that of the gelatin prepared by us (depending on the type of gelatin fraction and the preparation conditions it was 90–97%).

### 3.2. Discussion Summary

Previous studies devoted to the processing of MDCM by-products in gelatins used traditional methods of converting collagen to gelatin, consisting of the application of procedures commonly used in the industrial production of gelatins from beef and pork bones, first demineralizing of the starting raw material in an acidic environment, then conditioning the purified collagen in an alkaline environment, and eventually extracting the gelatins with hot water [42,43,44]. Taking into account the current obligations of the most economically developed countries in the world in terms of introducing environmentally friendly production procedures, we proposed a purely biotechnological method of preparing gelatin from MDCM by-product consisting of conditioning the defatted MDCM by-product followed by a 2-stage extraction of gelatin with hot water [41]. Although our technology, compared to the aforementioned studies that focused on obtaining gelatin from the MDCM by-product, was characterized by a much higher degree of conversion of the collagen raw material starting with gelatin (up to 32%), the quality of gelatin, represented primarily by its gel formation abilities, did not reach parameters that would predetermine them for use in some food and pharmaceutical products. For that reason, we designed a process to convert collagen into gelatin using the advantages of traditional and environmentally friendly procedures. After the separation of accompanying organic substances (albumins, globulins, and fat), the MDCM by-product is demineralized with 3.0% HCl, and purified collagen is conditioned with a proteolytic enzyme prior to gelatin hot-water extraction. This innovative and unique procedure leads to optimal disruption of the collagen quaternary and tertiary structure and the preparation of three qualitatively different gelatin fractions with a total yield of 55–77% (depending on the conditions of the process). With the appropriate choice of process conditions, high-quality gelatins can be prepared, which are fully comparable to gelatins produced by traditional methods from common collagen tissues. The Taguchi design, one of the designs of planned experiments (DOE), has proven to be an excellent tool for comprehensive process optimization in the processing of MDCM by-products into gelatins.

Although the MDCM by-product is an important and economically suitable raw material source for the production of gelatin, it is not used in industrial practice. On the one hand, there is limited information on suitable processing procedures and on the quality of the prepared gelatins, mainly consisting of determining the gel strength, viscosity, or the content of mineral substances. Our work provides a comprehensive overview of the gel-forming and surface properties of all three gelatin fractions. This information is important not only for the selection of gelatins for specific food, pharmaceutical, and medical applications but also for the choice of suitable processing technologies for final products containing gelatins.

Gelatins prepared from the first extraction fractions with zero gel strength and low viscosity but excellent EC and ES are suitable, for example, as an additive to meat products, improving the fat binding capacity and preserving the natural taste of the meat product. Furthermore, they can be used as an ingredient in the production of low-fat foods (cheese), in the production of fruit drinks to enhance sweet and fruity flavors, in the production of nutritional supplements, or in cosmetics as an ingredient in the production of cosmetic matrices (ointments and creams) for skin care. Most gelatins prepared from the second extraction fractions and some gelatins from the third extraction fractions stand out with excellent gel-forming properties and very good surface properties, which predisposes them to the most widespread food applications (gummy bears, jelly candies, marshmallows, or aspics).

The maceration liquor remaining after the demineralization of the raw material is a waste acid containing dissolved mineral components of bones in the form of phosphates formed according to the reactions:

Ca_3_(PO_4_)_2_ + 4 HCl → Ca(H_2_PO_4_)_2_ + 2 CaCl_2_ (1)

Ca_3_(PO_4_)_2_ + 6 HCl → 3 CaCl_2_ + 2 H_3_PO_4_ (2)

Ca_3_(PO_4_)_2_ + 4 H_3_PO_4_ → 3 Ca(H_2_PO_4_)_2_ (3)

This liquid by-product (phosphoric liquor) can be used to produce a precipitate, calcium hydrogen phosphate. A 10% solution of Ca(OH)_2_ is suitable for precipitation; the chemical reactions are as follows:

Ca(H_2_PO_4_)_2_ + Ca(OH)_2_ → 2 CaHPO_4_ + 2 H_2_O (4)

H_3_PO_4_ + Ca(OH)_2_ → CaHPO_4_ + 2 H_2_O (5)

The precipitate (white powder) contains approximately 76 % CaHPO_4_·2H_2_O, 20 % Ca_3_(PO_4_)_2_ and 4 % H_2_O; it is insoluble in water and soluble in weak acids. It can be used as an additive to feed mixtures for farm animals or as a fertilizer.

The undecomposed residue after gelatin extraction, which, depending on the process conditions, represents 23–46% of purified (demineralized) collagen, can be used as a secondary source of protein in feed mixtures for farm animals and pets, or due to its high protein content, as a source of nitrogen for the production of plant growth stimulators.

## 4. Materials and Methods

### 4.1. Materials, Appliances and Chemicals

Mechanically deboned chicken meat (MDCM) by-product (from Ross 708 broiler chicken aged 35 days) was supplied by Raciola, Ltd. (Uherský Brod, Czech Republic). First, by-product material analyses were performed by conventional food methods [50,51,52]. Dry matter content 38.2 ± 0.7%; in dry matter: protein 40.3 ± 1.2%, collagen (as a part of protein content) 79.9 ± 0.5%, fat 26.0 ± 1.5% and inorganic solids 29.6 ± 3.8%. Each analysis was repeated three times; mean values and standard deviations were calculated.

Stevens LFRA texture analyzer (Leonard Farnell and Co Ltd., Liverpool, UK), Ubbelohde viscometer (Technisklo Ltd., Držkov, Czech Republic), Nedform LT 43 shaker (Valašské Meziřící, Czech Republic), electronic scale Kern 440-47, electronic analytical balance Kern 770 (Balingen, Germany), analytical mill IKA A 10 labortechnik (Staufen, Germany), Memmert ULP 400 drying oven (Bűchenbach, Germany), Samsung fridge freezer (Seoul, Republic of Korea), Henkelman Boxer 42 vacuum packaging machine (CK ‘s-Hertogenbosch, Netherlands), IKA T 25 digital Ultra-Turrax (IKA-Werke, Germany), Whatman no. 1 paper (Sigma Aldrich, Gillingham, UK), WTW Multical pH 526 pH meter (Weilheim, Germany), heating board Schott Geräte (Mainz, Germany), a 1 mm pores size metal filter sieve (Labor-komplet, Praha, Czech Republic), ordinary laboratory glass.

Chemicals: NaCl, NaOH, HCl, petroleum ether, ethanol (Verkon, Prague, Czech Republic); all chemicals were analytical grade. Protamex^®^, Novozymes endopeptidase (Copenhagen, Denmark), used for conditioning purified collagen. It is a *Bacillus* protease complex with declared activity of 1.5 AU/g; optimal working conditions are at pH 5.5 to 7.5 and temperature 60 °C. The enzyme complies with the recommended purity specifications for food-grade enzymes issued by the Joint FAO/WHO Expert Committee on Food Additives (JECFA) and the Food Chemicals Codex (FCC).

### 4.2. Experimental Design and Statistical Analysis

Design of experiments (DOE) is a tool that enables the examination of the influence of independent variables (process factors) on dependent variables. Therefore, it enables the identification of significant factors of the process and its optimization [53]. Various experiment planning designs are used in practice, e.g., three-level full factorial design, central composite design, Box-Behnken design, or Taguchi design [54]. The extraction temperature and extraction time proved to be key process factors that influence not only the degree of conversion of collagen to gelatins but also the properties of gelatins. Therefore, these factors were studied using the Taguchi design of the experiments. This will achieve a more effective optimization of the gelatin preparation process from the MDCM by-product. Independent variables with factor levels: factor A (extraction temperature), 42, 46, 50 °C; factor B (extraction time), 20, 40, 60 min. The selected dependent variables were as follows: gelatin yields (Y_G1_, Y_G2_, Y_G3_), gel strength, viscosity, MP, GP, WHC, FBC, FC, FS, EC, and ES.

The gelatin analysis was performed in triplicate; mean values were calculated using Microsoft Office Excel 2013 (Microsoft, Denver, CO, USA). Minitab^®^ 17.2.1 statistical software for Windows (Fujitsu Ltd., Tokyo, Japan) was used to perform regression analysis of the data obtained. The statistical significance was evaluated using analysis of variance (ANOVA). The level of significance was established at 5% (*p*-value ≤ 0.05); factors with a value <0.05 have an effect on the process variables evaluated with 95% probability. The same software evaluated the graphical analysis of the data by creating contour plots showing the relationship between the dependent variables and the independent variables by viewing discrete contours of the dependent response variables.

### 4.3. Processing of MDCM By-Product into Gelatins

**Scheme 1:**
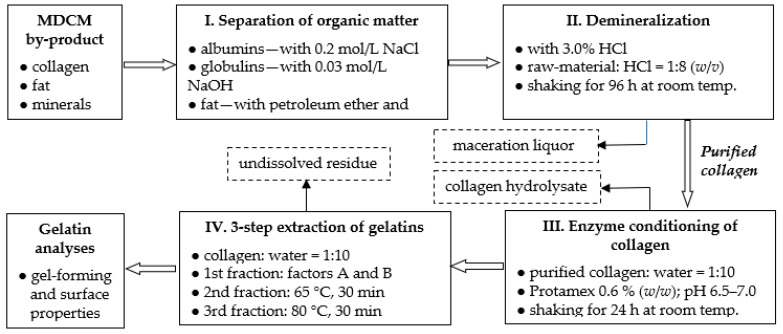
In our case, we proposed an innovative process of disrupting the quaternary structure of purified (demineralized) collagen: a proteolytic enzyme conditioning and gelatin extraction in 3 stages. The details of the procedure are given below.

The scheme of complex processing of MDCM by-product into three fractions of gelatins, including usable by-products created during processing, is shown in a flow chart in four technological sections, see Scheme 1.

I. Separation of organic matter. The thawed raw material was first washed with cold H_2_O. It was mixed with 0.2 mol/L NaCl in a 1: 6 ratio and shaken at room temperature (22.0 ± 1.0 °C) for 90 min and then washed with cold H_2_O. It was then mixed with 0.03 mol/L NaOH in a 1:6 ratio and shaken at room temperature for 45 min and, after filtration, washed with cold H_2_O; this procedure was repeated three more times. Finally, the raw material was washed with cold H_2_O and dried at 35 ° C for 24 h. This was followed by the defatting step: the raw material was mixed in a 1:9 (*w*/*v*) ratio with petroleum ether and ethanol (mixed in a ratio of 1:1, *v*/*v*) and shaken for 48 h at room temperature; after 12 h, the solvent was changed.

II. Demineralization. The raw material was mixed in a 1:8 ratio with 3.0% HCl and demineralized with gentle shaking at room temperature for 96 h; after 24 h, the acid was replaced with a new one. After filtration (maceration liquor as a by-product of the process), the demineralized collagen was thoroughly washed with cold H_2_O and dried for 24 h at 35 °C.

III. Purified collagen was mixed with H_2_O in a 1:10 ratio and after shaking for 20 min, the pH was adjusted to 6.5–7.0 (by adding a 5% NaOH solution). The 0.6 % proteolytic enzyme (based on the weight of purified collagen) was then added and the mixture was shaken at room temperature for 24 h; during the first 4 h at 30-min intervals, the pH was checked (and adjusted) to the prescribed range. After filtering off the liquid by-product (collagen hydrolysate), solid collagen was washed thoroughly with cold H_2_O. Collagen hydrolysate was dried in a thin layer (4 mm) in a circulating air drier at 60.0 ± 0.5 °C for 20 h.

IV. 3-step extraction of gelatins. Biotechnologically treated collagen was subjected to 3 separate (sequential) extraction cycles using a batch process extractor. In the first extraction stage, collagen was mixed with H_2_O in a ratio of 1:20 and the mixture was heated while stirring at a rate of dt/dτ = 10 °C/min to a temperature according to factor A (42.0 ± 0.5, 46.0 ± 0.5, 50.0 ± 0.5 °C), at which point the gelatin extraction lasted for the time according to factor B (20, 40, 60 min). After filtration, the solution of the 1st gelatin fraction was immediately heated to a temperature of 85.0 ± 0.5 °C (dt/dτ = 15 °C/min) and kept at this temperature for 8 min; the residual enzyme was inactivated this way. The gelatin solution was poured into a thin film (4 mm) and dried in a circulating air drier, first at 40.0 ± 0.5 °C for 12 h, and then at 65.0 ± 0.5 °C for 8 h. The resulting gelatin film was scraped, weighed, and ground to a powder. In the second and third extraction stages, the same procedure was followed at extraction temperatures of 65.0 ± 0.5 °C for 30 min and 80.0 ± 0.5 °C for 30 min. The second gelatin fraction was inactivated in the same way as the first gelatin fraction. The undissolved residue (a by-product of the extraction of gelatin) remained after the third extraction cycle and was dried at 103.0 ± 1.0 °C to constant weight and then weighed. The prepared gelatins were subjected to further analysis.

### 4.4. Analytical Part

The hydrolysate yield (Y_H_) was calculated from the weight of the hydrolysate prepared after conditioning the purified collagen according to the initial weight of the purified collagen (Equation (6)), the yield of gelatins (Y_G1_, Y_G2_, Y_G3_) from the weight of extracted gelatins according to the initial weight of the purified collagen (Equation (7)). Furthermore, the total extraction yield of gelatin (ΣY_G_) and the portion of undissolved residue (UR) was calculated (Equations (8) and (9)). The mass balance error (MBE) is expressed by the percentage difference of the dry matter mass balance between the input (purified collagen) and the output (hydrolysate + gelatins + undissolved residue); see Equation (10).

Y_H_ = (m_H_/m_0_) × 100 (6)

Y_G_ = (m_G_/m_0_) × 100 (7)

ΣY_G_ = Y_G1_+Y_G2_ + Y_G3_ (8)

UR = (m_UR_/m_0_) × 100 (9)

MBE = [100 − (Y_H_ + Y_G1_ + Y _G2_ + Y _G3_ + UR)] (10)

where Y_H_ is the hydrolysate yield (%), Y_G1_ is the yield of the first gelatin fraction (%), Y_G2_ is the yield of the second gelatin fraction (%), Y_G3_ is the yield of the third gelatin fraction (%), UR is an undissolved residue (%), m_0_ is the weight of purified collagen (g), m_H_ is the hydrolysate weight (g), m_G_ is the weight of gelatins (g), and m_UR_ is the weight of the undissolved residue (g).

Gel strength, viscosity, and ash content were determined according to standard test methods for edible gelatins [55]. Because these are common gelatin testing methods, we present only their principles. The gel strength was determined from a gel formed from a 6.67 % solution prepared according to prescribed conditions by measuring the force (weight in grams, which is equal to the Bloom value) required to depress a prescribed area of the sample surface to a distance of 4 mm. The viscosity of a 6.67 % gelatin solution was determined by the Ubbelohde viscometer and ash gravimetrically after burning and annealing the sample. The following gelatin properties are not described in standard gelatin testing methods, so a brief test procedure will be provided.

Gelatin water holding capacity was determined according to Nasrin et. al. [56] with slight modifications. In a plastic test tube, 1.0 g of the gelatin sample was mixed with 25.0 mL of distilled H_2_O and then the contents were shaken vigorously for 5 min at room temperature. The contents of the test tube were then centrifuged at 5000 rpm for 30 min and then the supernatant was filtered through Whatman no. 1. filter paper. WHC (%) was calculated from the weight of water absorbed by the gelatin sample, w_1_ (g), based on the weight of gelatin weighed, w_0_ (g), and multiplied by a coefficient of 100; see Equation (11).

WHC = (w_1_/w_0_) × 100 (11)

Gelatin fat binding capacity was determined according to Li et. al. [57]. In a plastic test tube, 0.1 g of the gelatin sample was mixed with 10.0 mL of sunflower oil, and the contents were vigorously shaken for 30 min at room temperature. The contents of the test tube were then centrifuged at 2500 rpm for 30 min and the supernatant was pipetted and weighed. FBC (%) was calculated from the weight of oil absorbed by the gelatin sample, w_2_ (g), based on the weight of gelatin weighed, w_0_ (g), and multiplied by a coefficient of 1000; see Equation (12).

FBC = (w_2_/w_0_) × 1000 (12)

Gelatin foaming capacity and foaming stability were determined according to Sathe et. al. [58] with slight modifications. The amount of 1.0 g of the gelatin sample was weighed in a graduated cylinder and 50.0 mL of distilled H_2_O was added; the gelatin was dissolved in a water bath at 60.0 ± 1.0 °C while stirring. After dissolving, a dispersing instrument was placed below the level of the resulting solution and the solution was whipped at 10,000 rpm for 5 min. After whipping, the volume of the whipped solution was measured; FC (%) was calculated according to Equation (13). After standing at room temperature for 30 min, the volume of the whipped solution was measured again; FS (%) was calculated according to Equation (14).

FC = [(V_1_ − V_0_)/V_0_] × 100 (13)

FS = [(V_2_ − V_0_)/V_0_] × 100 (14)

where V_0_ is the original volume of liquid (50 mL), V_1_ is the volume of the whipped solution (mL), and V_2_ is the volume of the whipped solution after 30 min (mL).

Gelatin emulsifying capacity and the emulsion stability were determined according to Neto et. al. [59] with slight modifications. In a plastic test tube, 0.01 g of the gelatin sample was mixed with 5.0 mL of distilled H_2_O, and after 10 s of thorough shaking, 5.0 mL of sunflower oil was added and shaken for 1 min at room temperature. The contents of the test tube were then centrifuged at 1000 rpm for 5 min. The heights of the entire volume of liquid in the tube and the emulsion were measured. The tube was then placed in a preheated water bath at 55.0 ± 0.5 °C for 5 min; then, the emulsion height was measured. EC (%) and ES (%) were calculated according to Equations (15) and (16).

EC = (h_1_/h_0_) × 100 (15)

ES = (h_2_/h_0_) × 100 (16)

where h_0_ is the height of the entire volume of liquid (mm), h_1_ is the height of the emulsion after centrifugation (mm), and h_2_ is the height of the emulsion after 5 min of heating (mm).

The Moosavi-Nasab method [60] with some modifications was used to determine the melting point; a solution of gelatin at the same concentration (6.67%) as after determination of gel strength and viscosity was used. A gelatin solution was introduced into a glass capillary of 3.0 mm in diameter to form a column at a height of 6.0 ± 1.0 mm. The sample capillary was allowed to cool at 10.0 ± 0.1 °C for 17 h (sol-gel transition). The capillary was then placed in a water bath at 10.0 ± 0.5 °C so it was completely immersed. The water bath was heated at 2 °C/min and the gelatin column in the capillary was monitored. The temperature at which the gelatin column began to move in the capillary (gel-sol transition) was recorded as the MP.

The Schrieber and Gareis method [2] with slight modifications was used to determine the gelling point; a gelatin solution at the same concentration (6.67%) as after determination of gel strength and viscosity was used. The gelatin solution in the test tube was placed in a water bath. After warming to 35.0 ± 0.5 °C, ice water was added to the water bath so that the cooling rate of the gelatin solution in the tube was 2 °C/min. Each time the temperature dropped by 0.5 °C, a 0.10 g metal ball was inserted into the tube. The temperature at which the ball got stuck in or on the gelatin solution layer was recorded as a GP.

## 5. Conclusions

The work is a contribution to the resolution of issues of environmental aspects of biomaterials. It has been proven that with the appropriate choice of innovative processing technology using Taguchi design as a modern method of experiment planning, it is possible to prepare high-quality gelatins from unused residue arising during the production of mechanically deboned chicken meat (MDCM). A completely new benefit of the work is the very high gelatin yields, which have not been achieved in previous works dealing with MDCM by-product processing. Furthermore, intermediate products formed during processing do not represent residual waste and can be further used; the presented technology belongs to zero waste processing of the MDCM by-product. One of the practical benefits of the work is that, even from a nontraditional source of collagen, gelatins of different quality can be prepared by multistage extraction. These are comparable to traditional pork and beef gelatins. Gelatins prepared from MDCM by-products are suitable for common food and pharmaceutical applications, for cosmetic products, and for production of biomedical matrixes as well.

## Figures and Tables

**Table 1:** The experimental design and the results of the process mass balance.

Exp. No.	Factor A (°C)	Factor B (min)	Y_H_ (%)	Y_G1_ (%)	Y_G2_ (%)	Y_G3_ (%)	UR (%)	MBE (%)	Y_G∑_ (%)
1	42	20	10.6	6.2	39.7	8.0	32.6	2.9	53.9
2	42	40	12.0	22.5	44.8	3.2	14.2	3.3	70.5
3	42	60	11.1	25.4	49.3	2.1	8.9	3.2	76.8
4	46	20	12.4	45.4	24.2	2.9	11.8	3.3	72.5
5	46	40	11.7	38.4	30.4	4.0	12.2	3.3	72.8
6	46	60	11.0	19.2	49.8	3.9	14.6	1.5	72.9
7	50	20	12.1	29.1	30.8	7.2	17.8	3.0	67.1
8	50	40	11.6	30.5	27.1	7.3	21.1	2.4	64.9
9	50	60	10.8	32.8	22.4	7.2	21.9	4.9	62.4
10 *	46	40	3.3	1.3	2.4	4.4	86.9	1.7	8.1

Factor A—extraction temperature at 1st extraction step; Factor B—extraction time at 1st extraction step; Y_H_—the yield of collagen hydrolysate; Y_G1_—the yield of the 1st gelatin fraction; Y_G2_—the yield of the 2nd gelatin fraction; Y_G3_—the yield of the 3rd gelatin fraction; UR—an undissolved residue; MBE—the mass balance error; Y_G**∑**_—total gelatin extraction yield; * Exp. No. 10—a blind experiment (no enzyme conditioning).

**Table 2:** Analysis of variance of the experimental design for gelatin yields.

	Degree of Freedom	Sum of Squares	Mean Squares	F-Value	*p*-Value
Response: The yield of the 1st gelatin fraction, Y_G1_ (%) = −44.6 + 1.60A − 0.028B					
Regression	2	246.30	123.148	0.94	0.441
Factor A (Extraction temperature)	1	244.48	244.482	1.87	0.220
Factor B (Extraction time)	1	1.82	1.815	0.01	0.910
Error	6	784.12	130.686		
Total	8	1030.42			
Response: The yield of the 2nd gelatin fraction, Y_G2_ (%) = 129.0 − 2.229A + 0.223B					
Regression	2	596.7	298.37	5.59	0.043
Factor A (Extraction temperature)	1	477.0	477.04	8.94	0.024 ^●^
Factor B (Extraction time)	1	119.7	119.71	2.24	0.185
Error	6	320.2	53.36		
Total	8	916.9			
Response: The yield of the 3rd gelatin fraction, Y_G3_ (%) = −9.4 + 0.350A − 0.0408B					
Regression	2	15.762	7.881	1.79	0.246
Factor A (Extraction temperature)	1	11.760	11.760	2.67	0.153
Factor B (Extraction time)	1	4.002	4.002	0.91	0.377
Error	6	26.407	4.401		
Total	8	42.169			

^●^ statistically significant factor (*p*-value ≤ 0.05).

**Table 3:** Results of the analysis of the properties of the first gelatin fractions.

Process Factors				Gelatin Properties						
Exp. No.	Factor A (°C)	Factor B (min)	Ash (%)	υ (mPa·s)	WHC (%)	FBC (%)	FC (%)	FS (%)	EC (%)	ES (%)
1	42	20	1.17	1.7	220	840	8	0	47	93
2	42	40	0.97	1.6	220	900	8	2	47	92
3	42	60	1.23	1.5	230	920	7	2	48	93
4	46	20	0.88	1.6	230	1090	6	2	48	93
5	46	40	0.96	1.5	230	1090	7	2	48	94
6	46	60	1.43	1.5	240	1110	7	2	47	93
7	50	20	1.39	1.5	240	1140	8	3	47	93
8	50	40	1.02	1.4	250	1210	8	4	48	95
9	50	60	1.16	1.4	240	1210	7	3	46	94
10 *	46	40	1.02	1.6	240	1150	7	4	48	93

Factor A—temperature at 1st extraction step; Factor B—extraction time at 1st extraction step; υ—viscosity; WHC—water holding capacity; FBC—fat binding capacity; FC—foaming capacity; FS—foaming stability; EC—emulsifying capacity; ES—emulsion stability; * Exp. No. 10—a blind experiment (no enzyme conditioning).

**Table 4:** Results of the analysis of the properties of the second gelatin fractions.

Process Factors						Gelatin Properties							
Exp. No.	Factor A (°C)	Factor B (min)	GS (Bloom)	MP (°C)	GP (°C)	υ (mPa·s)	Ash (%)	WHC (%)	FBC (%)	FC (%)	FS (%)	EC (%)	ES (%)
1	42	20	174	35.3	16.6	2.2	0.66	1010	1310	20	16	48	93
2	42	40	80	28.9	15.0	1.6	0.35	930	980	18	8	46	93
3	42	60	125	32.3	15.3	1.8	0.34	970	1390	18	10	47	93
4	46	20	143	32.8	15.5	1.9	0.43	950	1070	22	18	48	93
5	46	40	105	30.4	14.9	2.0	0.36	960	1170	20	8	51	90
6	46	60	262	36.8	17.1	2.9	0.45	960	1230	20	16	48	93
7	50	20	284	37.9	16.7	2.7	0.46	980	1250	20	16	47	93
8	50	40	269	35.1	16.4	2.6	0.43	990	1470	20	18	49	90
9	50	60	290	38.4	17.6	3.8	0.70	1090	1460	36	24	52	92
10 *	46	40	460	35.1	26.8	6.8	0.54	1320	1540	40	28	54	93

Factor A—temperature at 1st extraction step; Factor B—extraction time at 1st extraction step; GS—gel strength; MP—melting point; GP—gelling point; υ—viscosity; WHC—water holding capacity; FBC—fat binding capacity; FC—foaming capacity; FS—foaming stability; EC—emulsifying capacity; ES—emulsion stability; * Exp. No. 10—a blind experiment (no enzyme conditioning).

**Table 5:** Analysis of variance of the experimental design for gelatin gel strength, gelatin viscosity, melting point, and gelling point.

	Degree of Freedom	Sum of Squares	Mean Squares	F-value	*p*-Value
Response: Gel strength (Bloom) = −723 + 19.33A + 0.64B					
Regression	2	36,870.8	18,435.4	5.69	0.041
Factor A (Extraction temperature)	1	35,882.7	35,882.7	11.07	0.016 ^●^
Factor B (Extraction time)	1	988.2	988.2	0.30	0.601
Error	6	19,451.2	3241.9		
Total	8	56,322.0			
Response: Viscosity (mPa·s) = −4.89 + 0.1458A + 0.01417B					
Regression	2	2.5233	1.2617	5.98	0.037
Factor A (Extraction temperature)	1	2.0417	2.0417	9.68	0.021 ^●^
Factor B (Extraction time)	1	0.4817	0.4817	2.28	0.182
Error	6	1.2656	0.2109		
Total	8	3.7889			
Response: Meting point (°C) = 5.2 + 0.621A + 0.0125B					
Regression	2	37.3767	18.6883	2.21	0.191
Factor A (Extraction temperature)	1	37.0017	37.0017	4.37	0.082
Factor B (Extraction time)	1	0.3750	0.3750	0.04	0.840
Error	6	50.8322	8.4720		
Total	8	88.2089			
Response: Gelling point (°C) = 8.44 + 0.1583A + 0.0100B					
Regression	2	2.6467	1.3233	1.60	0.277
Factor A (Extraction temperature)	1	2.4067	2.4067	2.92	0.138
Factor B (Extraction time)	1	0.2400	0.2400	0.29	0.609
Error	6	4.9489	0.8248		
Total	8	7.5956			

^●^ statistically significant factor (*p*-value ≤ 0.05).

**Table 6:** Results of the analysis of the properties of the third gelatin fractions.

Process Factors						Gelatin Properties							
Exp. No.	Factor A (°C)	Factor B (min)	GS (Bloom)	MP (°C)	GP (°C)	υ (mPa·s)	Ash (%)	WHC (%)	FBC (%)	FC (%)	FS (%)	EC (%)	ES (%)
1	42	20	80	29.2	14.9	1.7	0.67	550	1190	7	0	48	95
2	42	40	82	30.1	15.0	1.7	0.35	560	1180	6	0	47	97
3	42	60	88	30.8	15.3	1.8	0.81	680	1220	8	0	48	97
4	46	20	0	NA	NA	1.4	0.96	220	1060	20	12	48	96
5	46	40	0	NA	NA	1.5	0.71	210	990	18	11	47	96
6	46	60	0	NA	NA	1.5	0.67	220	1040	20	12	48	95
7	50	20	223	33.9	16.0	2.4	0.59	680	1100	17	0	47	96
8	50	40	225	34.4	16.2	2.4	0.65	680	1120	16	0	48	96
9	50	60	230	34.8	16.5	2.6	0.48	730	1130	16	0	48	97
10*	46	40	245	34.1	15.2	2.4	0.63	910	1220	19	0	47	91

Factor A—temperature at 1st extraction step; Factor B—extraction time at 1st extraction step; GS—gel strength; MP—melting point; GP—gelling point; υ—viscosity; WHC—water holding capacity; FBC—fat binding capacity; FC—foaming capacity; FS—foaming stability; EC—emulsifying capacity; ES—emulsion stability; * Exp. No. 10—a blind experiment (no enzyme conditioning); NA—not applicable.

**Table 7:** Conditions for processing collagen raw materials into gelatins and gelatin yield.

Conditions for Processing Collagen Raw Material into Gelatins	Gelatin Yield (%)
Current study. Collagen tissue: MDCM by-product	53.9–76.8
Separation of non-collagenous matter: 0.2 mol/L NaCl, 0.03 mol/L NaOH; defatting: petroleum ether + ethanol (22 °C, 48 h); demineralization: 3.0% HCl (22 °C, 96 h); washing with H_2_O (22 °C); conditioning: protease (pH 6.5–7.0, 24 h, 22 °C); filtration; washing with H_2_O; 1st gelatin extraction: H_2_O, 42–50 °C, 40–60 min; filtration; 2nd gelatin extraction: H_2_O, 65 °C, 30 min; filtration; 3rd gelatin extraction: 80 °C, 30 min; gelatin drying: 40 °C, 12 h and 65 °C, 8 h.	
[41]. Collagen tissue: MDCM by-product	23.2–38.6
Separation of non-collagenous matter: H_2_O, 0.2 mol/L NaCl, 0.03 mol/L NaOH; defatting: lipolytic enzyme (22 °C, 48 h), petroleum ether + ethanol (22 °C, 20 h); conditioning: protease (pH 6.5–7.0, 24–72 h, 22 °C); filtration; washing with H_2_O; 1st gelatin extraction: H_2_O, 64–80 °C, 60–180 min; filtration; 2nd gelatin extraction: H_2_O, 90 °C, 120 min; filtration; gelatin drying: 50 °C, 48 h.	
[42]. Collagen tissue: MDCM by-product	1.6–16.9
Defatting: hexane; washing with H_2_O (22 °C); separation of non-collagenous matter: 1% NaCl (pH 10.5–10.7, 22 °C, 30 min); filtration; conditioning: 1.64–8.36% HCl (22 °C, 24 h); filtration; washing with H_2_O; neutralisation to pH 6–7; gelatin extraction: 53–87 °C, 2–12 h; ion exchange: Purolite C-100-E; gelatin drying: 40–42 °C.	
[43]. Collagen tissue: MDCM by-product	6.0–16.0
Defatting: H_2_O (35 °C); washing with H_2_O (25 °C); demineralization: 3.0% HCl (10 °C, 24 h); washing with H_2_O (22 °C); conditioning: 4.0% NaOH (22 °C, 72 h); filtration; washing with H_2_O (25 °C); neutralisation: H_3_PO_4_ (22 °C); gelatin extraction: 60–80 °C, 2–12 h, pH 4.0; filtration; centrifugation (22 °C, 30 min); gelatin freeze drying.	
[44]. Collagen tissue: MDCM by-product	6.0–15.0
Separation of non-collagenous matter: H_2_O (35 °C, 1 h); washing with H_2_O; demineralization: 3.0% HCl (10 °C, 24 h); washing with H_2_O; conditioning: 2.0–4.2% NaOH (22 °C, 48 h); washing with H_2_O; neutralisation: H_3_PO_4_ (22 °C, pH 4.0); washing with H_2_O; gelatin extraction: 60–82 °C, 50–250 min; filtration; centrifugation (22 °C, 30 min); gelatin drying: 40–42 °C.	
[45]. Collagen tissue: camel bone	8.5–25.3
Separation of non-collagenous matter: H_2_O (22 °C); demineralization: 1.5–6.0% HCl (22 °C, 24–120 h); filtration; washing with H_2_O; drying: 50 °C, 24 h; conditioning: 6.0% HCl (22 °C, 72 h); washing and neutralization: H_2_O (18 °C); gelatin extraction: 40–80 °C, 0.5–3.5 h, pH 1–7; gelatin freeze drying.	
[46]. Collagen tissues: tuna, shark and rohu skins	11.3–19.7
Washing with H_2_O (18 °C); separation of non-collagenous matter: 0.1 mol/L NaOH; washing and neutralization: H_2_O; conditioning: 0.2 mol/L CH3COOH (4 °C, 24 h); washing (neutralization): H_2_O; gelatin extraction: 45 °C, 12 h; fat separation; gelatin freeze drying.	
[47]. Collagen tissue: bovine heart	
Separation of non-collagenous matter: 0.5 mol/L NaOH, 22 °C, 30 min; neutralization: HCl; defatting: 10% butylalcohol, 22 °C; 1st gelatin extraction: H_2_O, 80 °C, 4–6 h; gelatin freeze drying (–60 °C). Conditioning: 0.5 mol/L CH_3_COOH + enzyme (100–200 mg/1 g of tissue): 22 °C, 24 h; neutralization: NaOH; 2nd gelatin extraction: H_2_O, 80 °C, 2 h; gelatin freeze drying (–60 °C).	7.0–11.0
	66.0–85.0
[8]. Collagen tissue: duck skin	11.7–44.0
Separation of non-collagenous matter: H_2_O (22 °C); conditioning: 0.1 mol/L HCl (18 °C, 24 h, pH 1.0); washing (neutralization): H_2_O (18 °C, 48 h); gelatin extraction (4 different methods): H_2_O (60 °C, 10 min), sonification in H_2_O (40 kHz, 60 °C, 10 min), steam (150 °C, 10 min), microwave (2450 MHz, 200 W, 10 min); filtration; gelatin coagulation (4 °C, 12 h); fat separation; gelatin freeze drying (–40 °C).	
[18]. Collagen tissue: tuna skin	11.3
Separation of non-collagenous matter and pigment: H_2_O (40 °C, 10 min), 0.1 mol/L NaOH (22 °C, 1 h); washing with H_2_O (18 °C); conditioning: 0.2 mol/L CH_3_COOH (4 °C, 12 h); washing and neutralization: H_2_O (45 °C, 12 h); gelatin extraction: 45 °C, 12 h; filtration; concentration to 15% dry matter (vacuum, 45 °C); gelatin freeze drying (–25 °C).	
Collagen tissue: frog skin	7.1–15.4
Separation of non-collagenous matter: 0.2 mol/L NaOH (4 °C, 30 min); washing: H_2_O (18 °C); conditioning: 0.05 mol/L CH_3_COOH (25 °C, 3 h); washing and neutralization: H_2_O (45 °C, 12 h); gelatin extraction: 45 °C, 12 h; filtration; concentration to 15% dry matter (vacuum, 45 °C); gelatin freeze drying (–25 °C).	
Collagen tissue: chicken skin	2.2
Defatting: 30% isopropylalcohol (22 °C, 2 h); separation of non-collagenous matter: 1.0% NaCl (22 °C, 30 min, pH 10.6); filtration; conditioning: 5.0% HCl (22 °C, 24 h); washing with H_2_O, neutralization (pH 7); gelatin extraction: 45–65 °C, 15 h; filtration; gelatin freeze drying (–25 °C).	

## Data Availability

The data are available from the corresponding author.

## References

[1] Djagny K.B., Wang Z., Xu S. (2001). Gelatin: A valuable protein for food and pharmaceutical industries, review. Crit. Rev. Food Sci. Nutr..

[2] Schrieber R., Gareis H. (2007). Gelatine Handbook—Theory and Industrial Practice.

[3] Saha D., Bhattacharya S. (2010). Hydrocolloids as thickening and gelling agents in food: A critical review. J. Food Sci. Technol..

[4] (2019). Gelatin Market Analysis.

[5] Abedinia A., Nafchi A.M., Sharifi M., Ghalambor P., Oladzadabbasabadi N., Ariffin F., Huda N. (2020). Poultry gelatin: Characteristics, developments, challenges, and future outlooks as a sustainable alternative for mammalian gelatin. Trends Food Sci. Technol..

[6] Choe J., Kim H.Y. (2018). Effects of chicken feet gelatin extracted at different temperatures and wheat fiber with different particle sizes on the physicochemical properties of gels. Poult. Sci..

[7] Sarbon N.M., Nazlin F.B., Howell K. (2013). Preparation and characterisation of chicken skin gelatin as an alternative to mammalian gelatin. Food Hydrocoll..

[8] Kim T.K., Ham Y.K., Shin D.M., Kim H.W., Jang H.W., Kim Y.B., Choi Y.S. (2020). Extraction of crude gelatin from duck skin: Effects of heating methods on gelatin yield. Poult. Sci..

[9] Abedinia A., Ariffin F., Huda N., Nafchi A.M. (2017). Extraction and characterization of gelatin from the feet of Pekin duck (Anas platyrhynchos domestica) as affected by acid, alkaline, and enzyme pretreatment. Int. J. Biol. Macromol..

[10] Saenmuang S., Phothiset S., Chumnanka C. (2020). Extraction and characterization of gelatin from black-bone chicken by-products. Food Sci. Biotechnol..

[11] Yuliani D., Awalsasi D., Jannah A. (2019). Characterization of gelatin profile of chicken broiler (*Gallus domestica*) bone using SDS-PAGE electrophoresis. Alchemy–J. Chem..

[12] Du L., Khiari Z., Pietrasik Z., Betti M. (2013). Physicochemical and functional properties of gelatins extracted from turkey and chicken heads. Poult. Sci..

[13] Zhang T., Sun R., Ding M., Tao L., Liu L., Tao N., Wang X., Zhong J. (2020). Effect of extraction methods on the structural characteristics, functional properties, and emulsion stabilization ability of tilapia skin gelatins. Food Chem..

[14] Chandra M.V., Shamasundar B.A. (2015). Rheological properties of gelatin prepared from the swim bladders of freshwater fish Catla catla. Food Hydrocoll..

[15] Akagündüz Y., Mosquera M., Giménez B., Alemán A., Montero P., Gómez-Guillén M.C. (2014). Sea bream bones and scales as a source of gelatin and ACE inhibitory peptides. LWT-Food Sci. Technol..

[16] Liu H.Y., Han J., Guo S.D. (2009). Characteristics of the gelatin extracted from Channel Catfish (*Ictalurus Punctatus*) head bones. LWT-Food Sci. Technol..

[17] Jamilah B., Tan K.W., Umi Hartina M.R., Azizah A. (2011). Gelatins from three cultured freshwater fish skins obtained by liming process. Food Hydrocoll..

[18] Tümerkan E.T.A., Cansu Ü., Boran G., Regenstein J.M., Özoğul F. (2019). Physiochemical and functional properties of gelatin obtained from tuna, frog and chicken skins. Food Chem..

[19] Norziah M.H., Al-Hassan A., Khairulnizam A.B., Mordi M.N., Norita M. (2009). Characterization of fish gelatin from surimi processing wastes: Thermal analysis and effect of transglutaminase on gel properties. Food Hydrocoll..

[20] Sinthusamran S., Benjakul S., Kishimura H. (2014). Characteristics and gel properties of gelatin from skin of seabass (*Lates calcarifer*) as influenced by extraction conditions. Food Chem..

[21] Jridi M., Nasri R., Lassoued I., Souissi N., Mbarek A., Barkia A., Nasri M. (2013). Chemical and biophysical properties of gelatins extracted from alkali-pretreated skin of cuttlefish (*Sepia officinalis*) using pepsin. Food Res. Int..

[22] Badway H.M.R., Abd El-Moniem S.M., Soliman A.M., Rabie M.A. (2019). Physicochemical properties of gelatin extracted from Nile tilapia (*Oreochromis niloticus*) and Nile perch (*Lates niloticus*) fish skins. Zagazig J. Agric. Res..

[23] Peterková P., Lapčík L. (2000). Collagen—Properties, modifications and applications. Chem. Listy.

[24] Ma Y., Zeng Y., Ma X., Yang R., Zhao W. (2019). A simple and eco-friendly method of gelatin production from bone: One-step biocatalysis. J. Clean. Prod..

[25] Pereira A.G.T., Ramos E.M., Teixeira J.T., Cardoso G.P., Ramos A.L.S., Fontes P.R. (2011). Effects of the addition of mechanically deboned poultry meat and collagen fibers on quality characteristics of frankfurter-type sausages. Meat Sci..

[26] Yuste J., Mor-Mur M., Capellas M., Guamis B., Pla R. (1999). Mechanically recovered poultry meat sausages manufactured with high hydrostatic pressure. Poult. Sci..

[27] Viuda-Martos M., Fernández-López J., Pérez-Álvarez J.A., Hui Y.H., Aalhus J.L., Cocolin L., Guerrero-Legarreta I., Nollet L.M., Purchas R.W., Schilling M.W., Stanfield P., Xiong Y.L. (2012). Mechanical Deboning. Handbook of Meat and Meat Processing.

[28] Daros F.G., Masson M.L., Amico S.C. (2005). The influence of the addition of mechanically deboned poultry meat on the rheological properties of sausage. J. Food Eng..

[29] Massingue A.A., de Almeida Torres F.R., Fontes P.R., de Lemos S.R.A., Fontes E.A.F., Perez J.R.O., Ramos E.M. (2018). Effect of mechanically deboned poultry meat content on technological properties and sensory characteristics of lamb and mutton sausages. Asian-Australas J. Anim. Sci..

[30] Cheow C.S., Norizah M.S., Kyaw Z.Y., Howell N.K. (2007). Preparation and characterisation of gelatins from the skins of sin croaker (*Johnius dussumieri*) and shortfin scad (*Decapterus macrosoma*). Food Chem..

[31] Gómez-Guillén M.C., Turnay J., Fernández-Díaz M.D., Ulmo N., Lizarbe M.A., Montero P. (2002). Structural and physical properties of gelatin extracted from different marine species: A comparative study. Food Hydrocoll..

[32] Eysturskarð J., Haug I.J., Elharfaoui N., Djabourov M., Draget K.I. (2009). Structural and mechanical properties of fish gelatin as a function of extraction conditions. Food Hydrocoll..

[33] Lin L., Regenstein J.M., Lv S., Lu J., Jiang S. (2017). An overview of gelatin derived from aquatic animals: Properties and modification. Trends Food Sci. Technol..

[34] Ogawa M., Portier R.J., Moody M.W., Bell J., Schexnayder M.A., Losso J.N. (2004). Biochemical properties of bone and scale collagens isolated from the subtropical fish black drum (*Pogonia cromis*) and sheepshead seabream (*Archosargus probatocephalus*). Food Chem..

[35] Yang H., Wang Y. (2009). Effects of concentration on nanostructural images and physical properties of gelatin from channel catfish skins. Food Hydrocoll..

[36] Haug I.J., Draget K.I., Smidsrød O. (2004). Physical and rheological properties of fish gelatin compared to mammalian gelatin. Food Hydrocoll..

[37] Jamilah B., Harvinder K.G. (2002). Properties of gelatins from skins of fish—Black tilapia (*Oreochromis mossambicus*) and red tilapia (*Orchromis nilotica*). Food Chem..

[38] Saxena A., Sachin K., Bohidar H.B., Verma A.K. (2005). Effect of molecular weight heterogeneity on drug encapsulation efficiency of gelatin nano-particles. Colloids Surf. B Biointerfaces.

[39] Muyonga J.H., Cole C.G.B., Duodu K.G. (2004). Fourier transform infrared (FTIR) spectroscopic study of acid soluble collagen and gelatifrom skins and bones of young and adult Nile perch (*Lates niloticus*). Food Chem..

[40] Badii F., Howell N.K. (2006). Fish gelatin: Structure, gelling properties and interaction with egg albumen proteins. Food Hydrocoll..

[41] Mokrejš P., Gál R., Pavlačková J., Janáčová D. (2021). Valorization of a by-product from the production of mechanically deboned chicken meat for preparation of gelatins. Molecules.

[42] Rafieian F., Keramat J., Kadivar M. (2011). Optimization of gelatin extraction from chicken deboner residue using RSM method. J. Food Sci. Technol..

[43] Rammaya K., Ying V.Q., Babji A.S. (2012). Physicochemical analysis of gelatin extracted from mechanically deboned chicken meat (mdcm) residue. Int. J. Food Saf. Nutr. Publ. Health.

[44] Erge A., Zorba Ö. (2018). Optimization of gelatin extraction from chicken mechanically deboned meat residue using alkaline pre-treatment. LWT-Food Sci. Technol..

[45] Al-Kahtani H.A., Jaswir I., Ismail E.A., Ahmed M.A., Monsur Hammed A., Olorunnisola S., Octavianti F. (2016). Structural characteristics of camel-bone gelatin by demineralization and extraction. Int. J. Food Prop..

[46] Shyni K., Hema G.S., Ninan G., Mathew S., Joshy C.G., Lakshmanan P.T. (2014). Isolation and characterization of gelatin from the skins of skipjack tuna (*Katsuwonus pelamis*), dog shark (*Scoliodon sorrakowah*), and rohu (*Labeo rohita*). Food Hydrocoll..

[47] Roy B.C., Das C., Hong H., Betti M., Bruce H.L. (2017). Extraction and characterization of gelatin from bovine heart. Food Biosci..

[48] European Pharmacopoeia 10.0 (2019). European Directorate for the Quality of Medicines & Health Care, Strasbourgh, France. https://www.scribd.com/document/508063535/European-Pharmacopoeia-10-0#.

[49] Food Chemical Codex 12. https://www.foodchemicalscodex.org/.

[50] Nollet L.M.L., Toldrá F. (2015). Handbook of Food Analysis.

[51] Meat and Meat Products—Determination of Hydroxyproline Content. https://cdn.standards.iteh.ai/samples/8848/908d030b1d6a4807bc2ac15fee8d51f9/SIST-ISO-3496-1995.pdf.

[52] Vázquez-Ortiz F.A., González-Méndez N.F. (1996). Determination of collagen as a quality index in Bologna from Northwestern Mexico. J. Food Compos. Anal..

[53] Antony J. (2014). Design of Experiments for Engineers and Scientists.

[54] Zhang J.Z., Chen J.C., Kirby E.D. (2007). Surface roughness optimization in an end-milling operation using the Taguchi design method. J. Mater. Process. Technol..

[55] Standard Testing Methods for Edible Gelatin Official Procedure of the Gelatin Manufacturers Institute of America, Inc. http://www.gelatin-gmia.com/images/GMIA_Official_Methods_of_Gelatin_Revised_2013.pdf.

[56] Nasrin T.A.A., Noomhorm A., Anal A.K. (2015). Physico-chemical characterization of culled plantain pulp starch, peel starch and flour. Int. J. Food Prop..

[57] Li F., Jia D., Yao K. (2009). Amino acid composition and functional properties of collagen polypeptide from yak (*Bos grunniens*) bone. LWT-Food Sci. Technol..

[58] Sathe S.K., Deshpande S.S., Salunkhe D.K. (1982). Functional properties of lupin seed (*Supinus mutabilis)* proteins and protein concentrates. J. Food Sci..

[59] Neto V.Q., Narain N., Silva J.B., Bora P.S. (2001). Functional properties of raw and heat processed cashew nut (*Anarcardium occidentale* L.) kernel protein isolates. Die Nahrung.

[60] Moosavi-Nasab M., Yazdani-Dehnavi M., Mirzapour-Kouhdasht A. (2020). The effects of enzymatically aided acid-swelling process on gelatin extracted from fish by- products. Food Sci. Nutr..

